# Conservative Management of a Complicated Crown-Root Fracture Using Fiber Post Reattachment: A Case Report

**DOI:** 10.7759/cureus.90246

**Published:** 2025-08-16

**Authors:** Omer Ozkilic, Gamze Durak Ozkilic

**Affiliations:** 1 Endodontics, South Cliff Dental, Horsham, GBR; 2 Endodontics, Dicle University, Diyarbakir, TUR

**Keywords:** complicated crown-root fracture, endodontics conservative dentistry, endodontic treatment, fiber reinforced post, management of dental trauma, maxillary central incisor

## Abstract

Dental trauma is frequently encountered among young adult males and may result in complex injuries involving both esthetic and functional challenges. This report describes the conservative treatment of a complicated crown-root fracture affecting the maxillary central incisor of a 24-year-old soldier who sustained facial trauma during military duty. The patient presented with swelling, bleeding, and a fractured anterior tooth. Initial management at a military hospital involved temporary stabilization of the mobile fragment. Upon clinical and radiographic examination, the fracture was initially believed to be limited to the coronal structure; however, removal of the fragment revealed an extension into the root with a 2 mm palatal periodontal pocket, confirming a diagnosis of complicated crown-root fracture. Root canal treatment was performed, followed by the placement of a fiber post. The preserved crown fragment, stored in saline, was reattached using adhesive bonding techniques. This approach provided favorable esthetic and functional outcomes while avoiding surgical intervention. The case emphasizes the importance of accurate diagnosis and individualized planning in the management of dental trauma. When appropriate, reattachment of the original fragment using fiber post support may serve as a reliable alternative to extraction or prosthetic replacement.

## Introduction

Traumatic dental injuries (TDIs) are relatively common in young adults, particularly males, and frequently occur during sports, traffic accidents, or occupational activities [1]. These injuries can result in significant esthetic, functional, and psychological consequences, especially when affecting the anterior region. Epidemiological studies report that crown-root fractures account for approximately 5% of all traumatic dental injuries, with a higher prevalence among young males [2].

Among TDIs, crown-root fractures involve enamel, dentin, and the root surface, often extending subgingivally, which complicates both diagnosis and treatment [3]. These fractures are classified according to pulp involvement (complicated vs. uncomplicated) and the location of the fracture line relative to the gingival margin [4]. According to the widely used Ellis and Davey classification, the present case corresponds to a Class III fracture, involving enamel, dentin, and pulp with subgingival extension [5].

Treatment options for complicated crown-root fractures include surgical crown lengthening, orthodontic extrusion, post-and-core restorations, or extraction followed by prosthetic rehabilitation or implant placement. In recent years, fragment reattachment supported by a fiber post has emerged as a conservative approach that preserves natural tooth structure while meeting esthetic and functional demands [6].

This case report presents the conservative and successful management of a complicated crown-root fracture in a young male soldier using fiber post-supported reattachment, discussing diagnostic challenges, treatment rationale, and its relevance within the current literature.

## Case presentation

A 24-year-old male soldier sustained orofacial trauma during military service after tripping and hitting his face on a rock. He initially presented to a military hospital with swelling, upper lip bleeding, and sensitivity in an anterior tooth. The mobile coronal fragment was temporarily reattached at the hospital. Approximately 24 hours later, he was referred to the Department of Endodontics at Dicle University Faculty of Dentistry.

**Figure 1:**
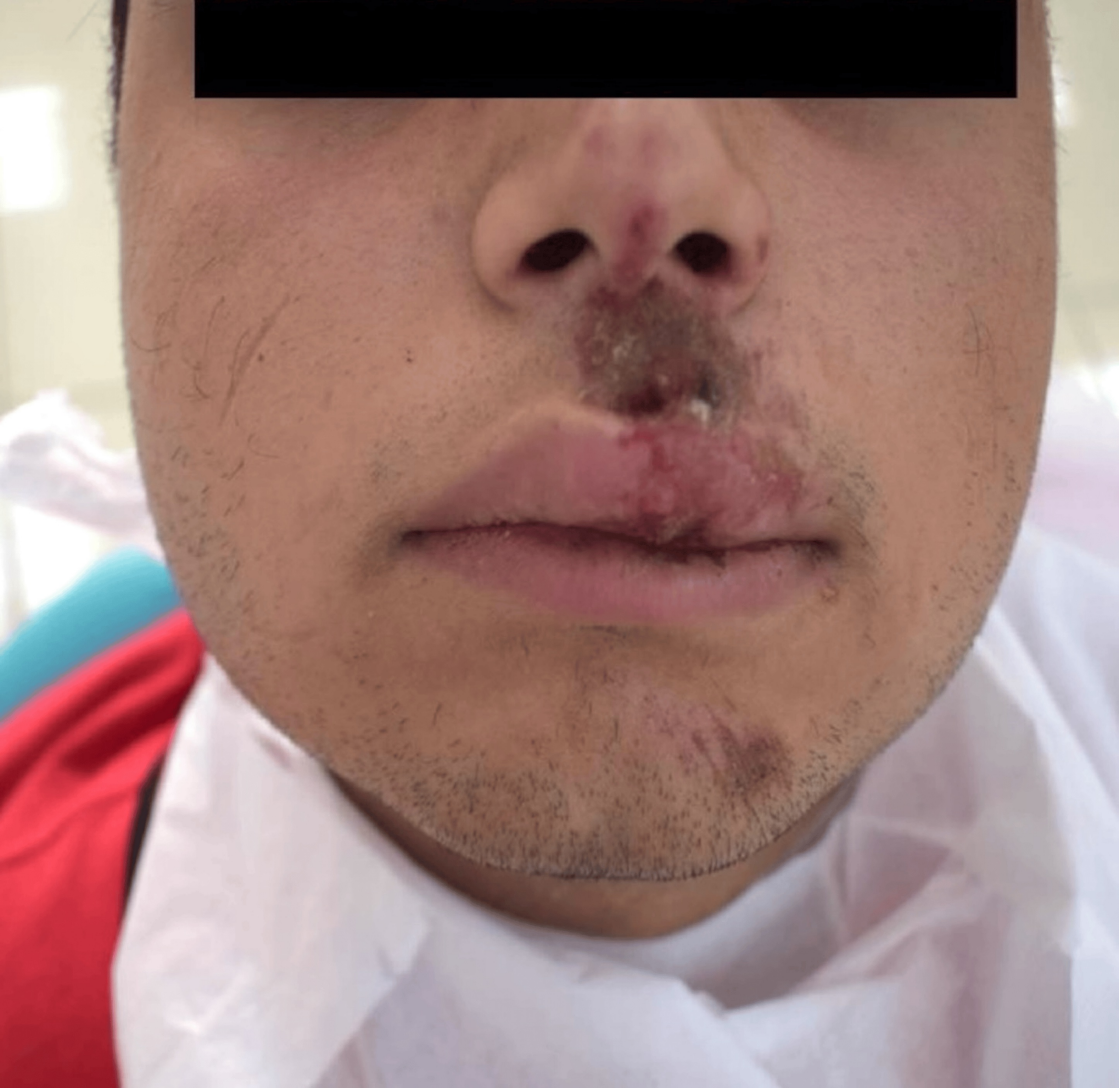
Extraoral view showing upper lip trauma Crusted hemorrhagic lesions and edema were noted on the upper lip, consistent with blunt facial trauma during military activity.

**Figure 2:**
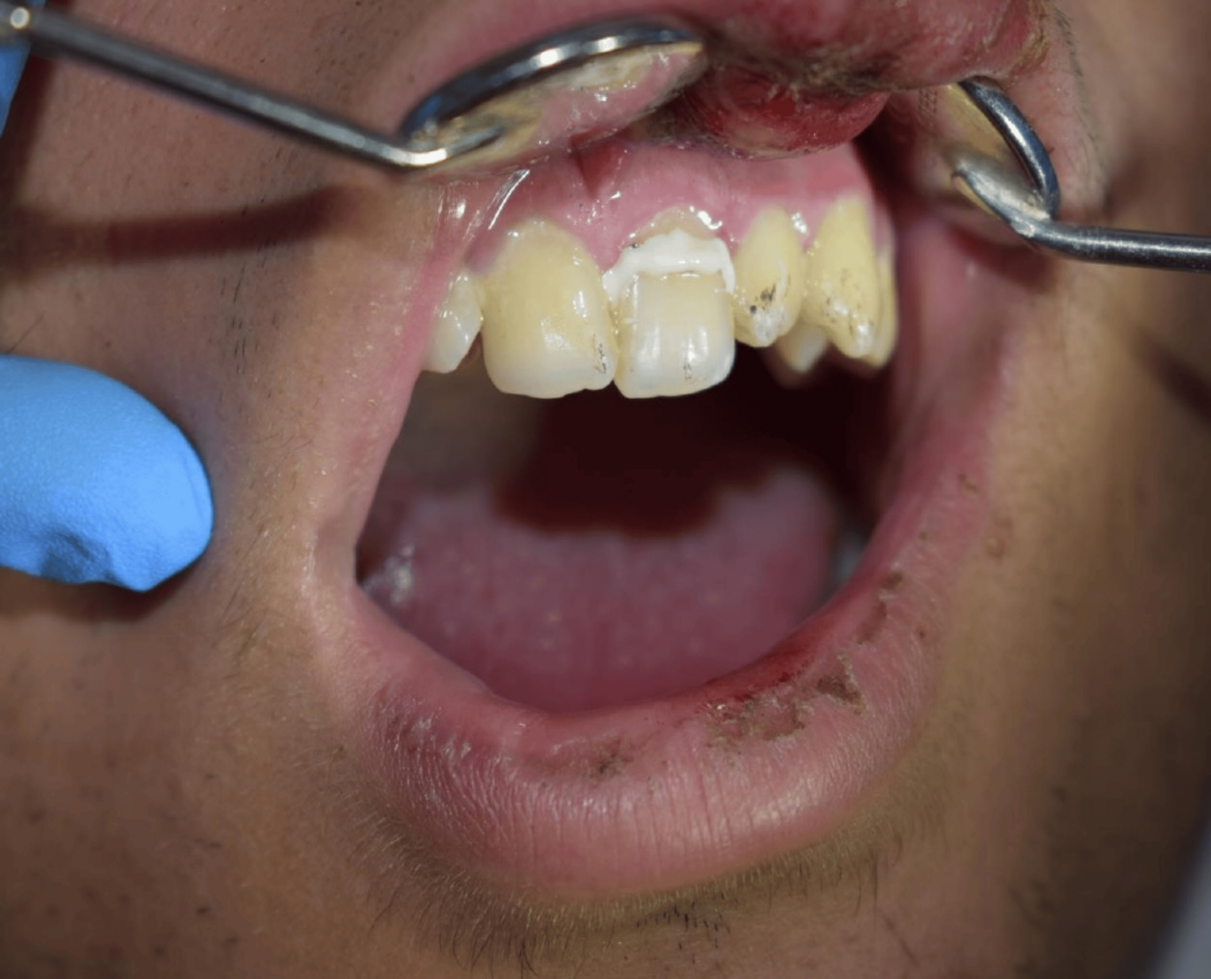
Intraoral view of tooth #21 with stabilized coronal fragment. The fractured coronal fragment of tooth #21 was temporarily stabilized at the military hospital prior to referral.

**Figure 3:**
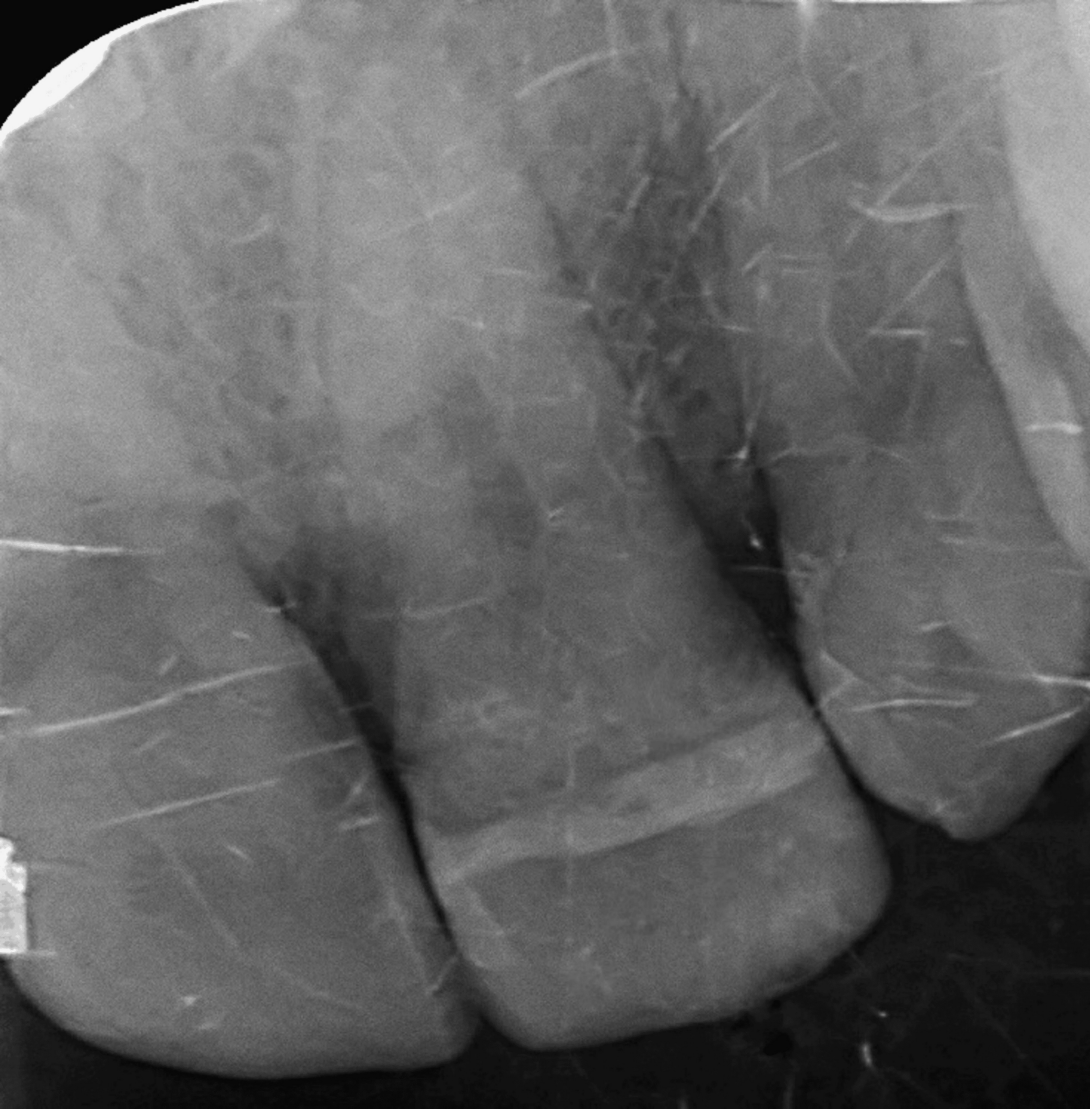
Preoperative periapical radiograph. Radiographic image suggesting a fracture line limited to the coronal portion of tooth #21, leading to an initial diagnosis of complicated crown fracture.

Extraoral examination revealed crusted hemorrhagic lesions and edema of the upper lip, consistent with facial trauma (Figure 1). Intraoral examination showed a mobile coronal fragment involving tooth #21, which had been temporarily stabilized (Figure 2). Periapical radiograph initially suggested that the fracture was limited to the coronal portion and did not appear to involve the pulp (Figure 3). Therefore, a preliminary diagnosis of uncomplicated crown fracture was considered.

**Figure 4:**
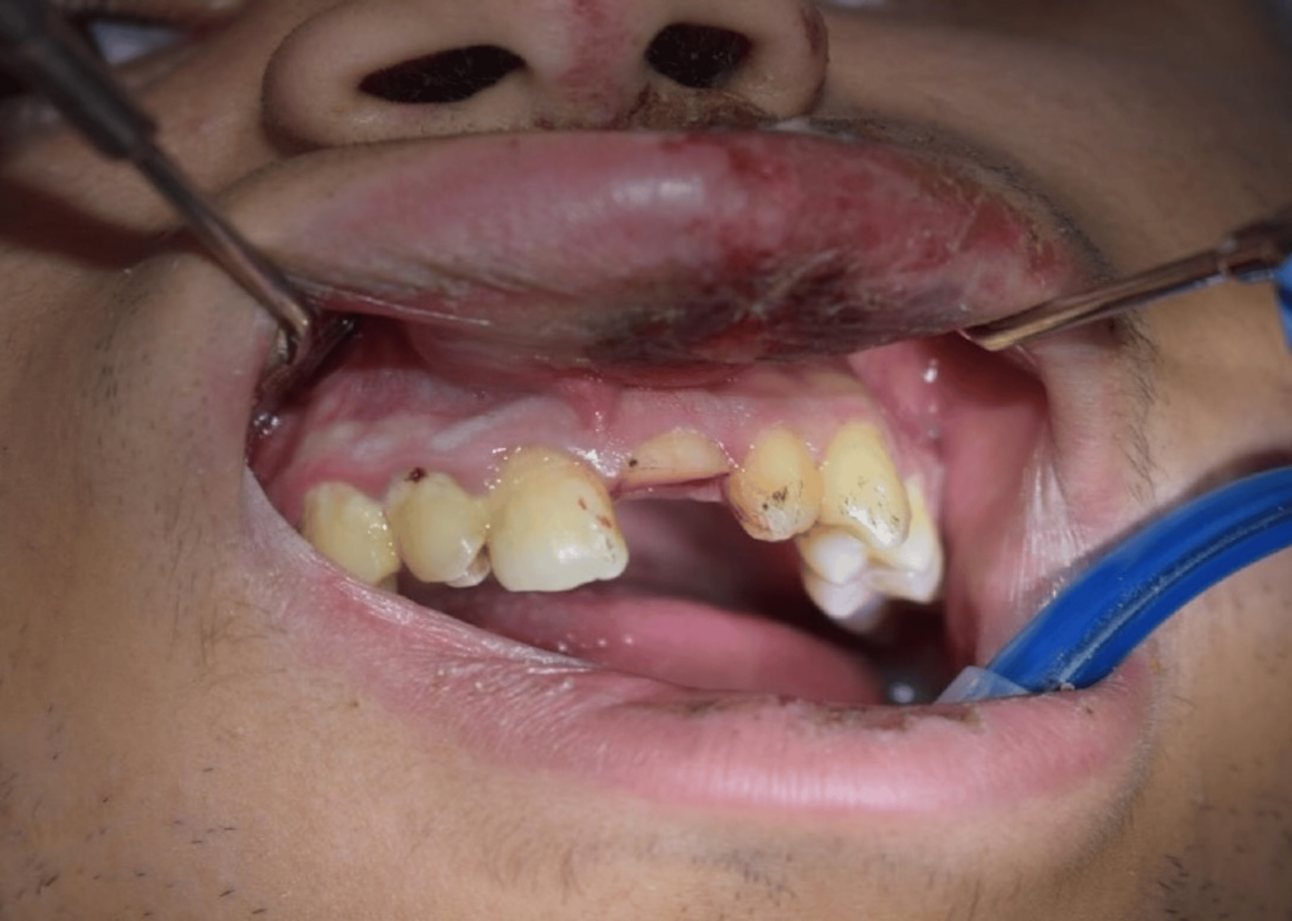
Post-removal view of coronal fragment Intraoral view after extraction of the mobile crown segment, confirming subgingival extension of the fracture.

**Figure 5:**
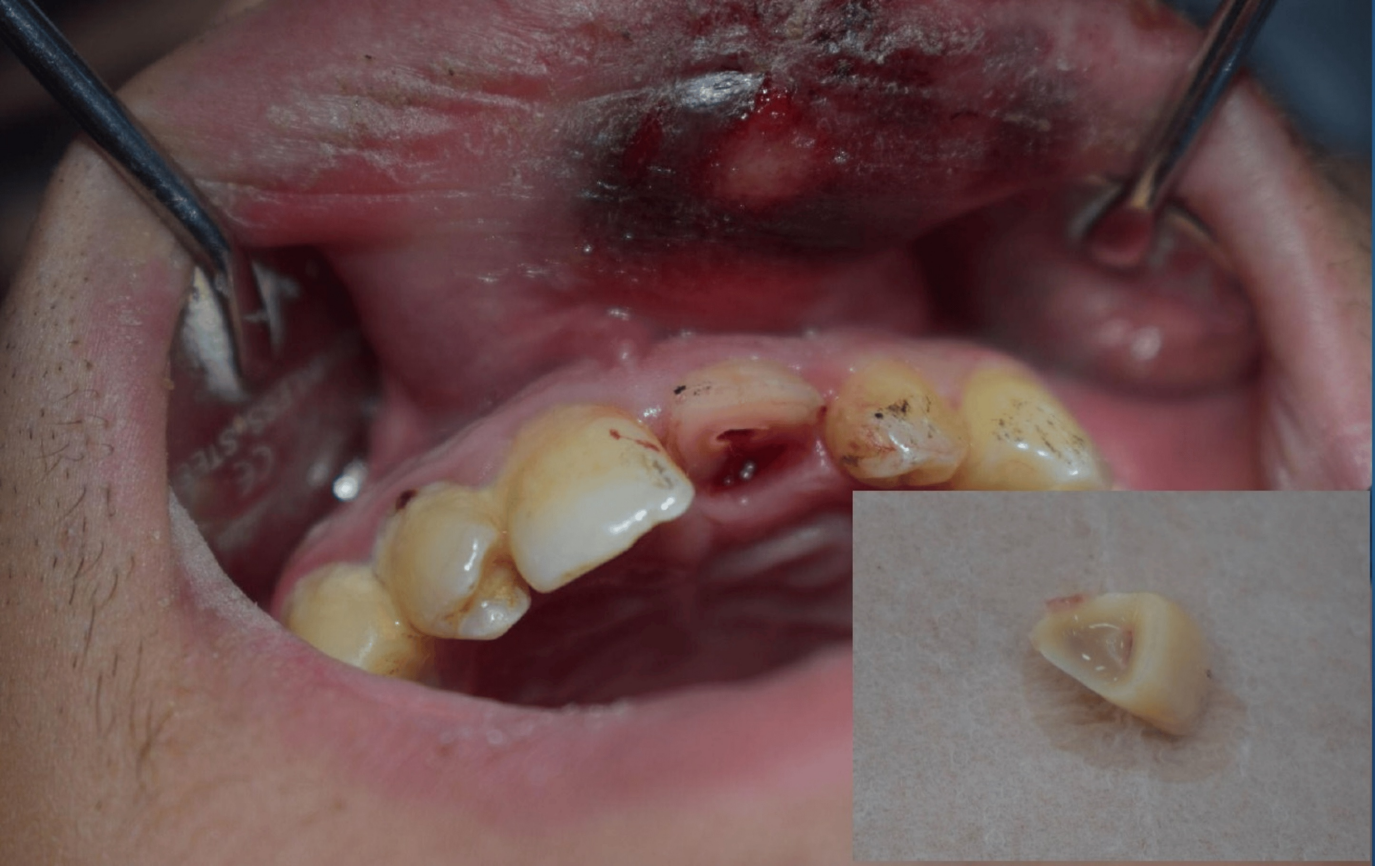
Assessment of fracture depth and extracted crown fragment Clinical image showing the fractured tooth with a 2-mm palatal probing depth (physiological range) and the extracted fragment preserved in sterile saline (inset). Note: Palatal aspect image was not captured due to clinical constraints, which is acknowledged as a limitation in the text.

However, following local anesthesia, the mobile coronal fragment was carefully removed. At this stage, it became evident that the fracture extended below the gingival margin onto the root surface (Figure 4). Further inspection revealed a palatal probing depth of approximately 2 mm, indicating a subgingival fracture extension. The definitive diagnosis was revised to a complicated crown-root fracture (Figure 5).

At this stage, alternative treatment options - including surgical crown lengthening, orthodontic extrusion, or extraction with prosthetic replacement - were considered. Given the limited subgingival extension, healthy periodontal support, and the patient’s preference to retain his natural tooth, a conservative reattachment approach was chosen.

The coronal fragment was stored in sterile saline, and root canal treatment was initiated. Access cavity preparation was performed using a high-speed diamond bur (Mani, Utsunomiya, Japan) under water cooling. Working length was determined using an electronic apex locator (Root ZX II; J. Morita, Suita, Japan) and confirmed radiographically. Biomechanical preparation was performed with rotary NiTi instruments (Reciproc R25, 25/.08; VDW, Munich, Germany) according to the manufacturer’s instructions. Irrigation was carried out using 2.5% sodium hypochlorite solution (Werax, Izmir, Turkey), approximately 20 mL in total, delivered with a side-vented irrigation needle (NaviTip; Ultradent, South Jordan, UT, USA). Final irrigation included 17% EDTA (Werax) for one minute, followed by a sterile distilled water rinse. The canal was dried with sterile paper points (Diadent, Cheongju, Korea) and medicated with a calcium hydroxide paste (Calcipast; Cerkamed, Stalowa Wola, Poland) using a Lentulo spiral. The access cavity was sealed with a temporary restorative material (Cavit-G; 3M ESPE, Seefeld, Germany).

**Figure 6:**
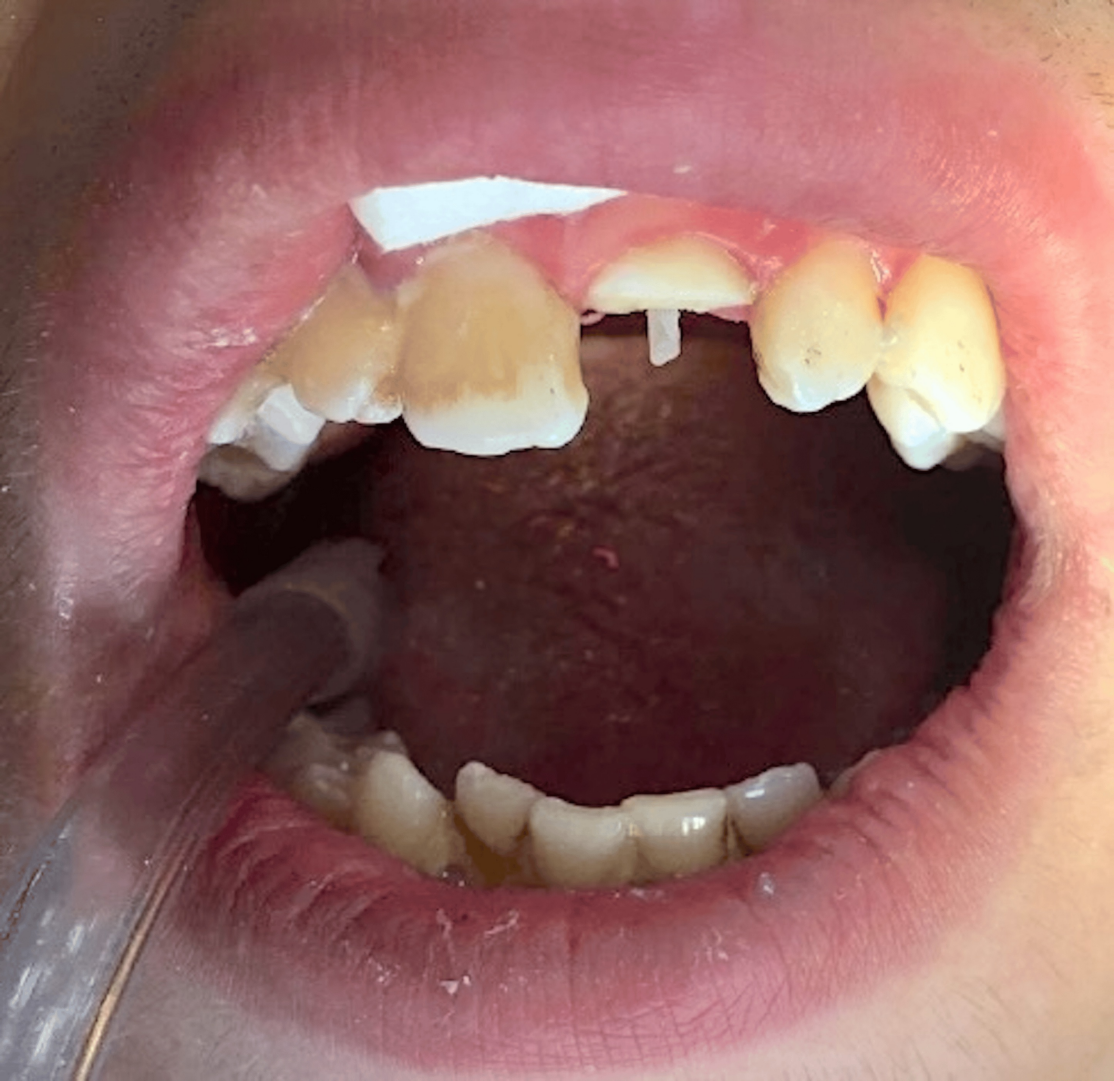
Fiber post placement Intraoral view during the second visit after obturation and post space preparation. A fiber post was positioned into the canal in the same session.

At the second visit, the intracanal medicament was removed, and the canal was obturated. Post space preparation was performed, and a fiber post (RelyX Fiber Post #2; 3M ESPE) was trial-fitted (Figure 6). A cavity was prepared in the internal surface of the coronal fragment to accommodate the post. Both the post space and fragment cavity were etched with 37% phosphoric acid (Scotchbond Universal Etchant; 3M ESPE) for 15 seconds, rinsed, and gently air-dried. A universal adhesive (Single Bond Universal; 3M ESPE) was applied, air-thinned, and light-cured with an LED curing unit (Bluephase N; Ivoclar Vivadent, Schaan, Liechtenstein) for 20 seconds. The fiber post was cemented with dual-cure resin cement (RelyX Ultimate; 3M ESPE) following the manufacturer’s instructions. The fragment was seated with firm finger pressure, excess resin removed, and light curing was performed for 40 seconds from buccal and palatal aspects. Final finishing and polishing were performed using fine-grit diamond burs (Mani) and polishing discs (Sof-Lex; 3M ESPE).

**Figure 7:**
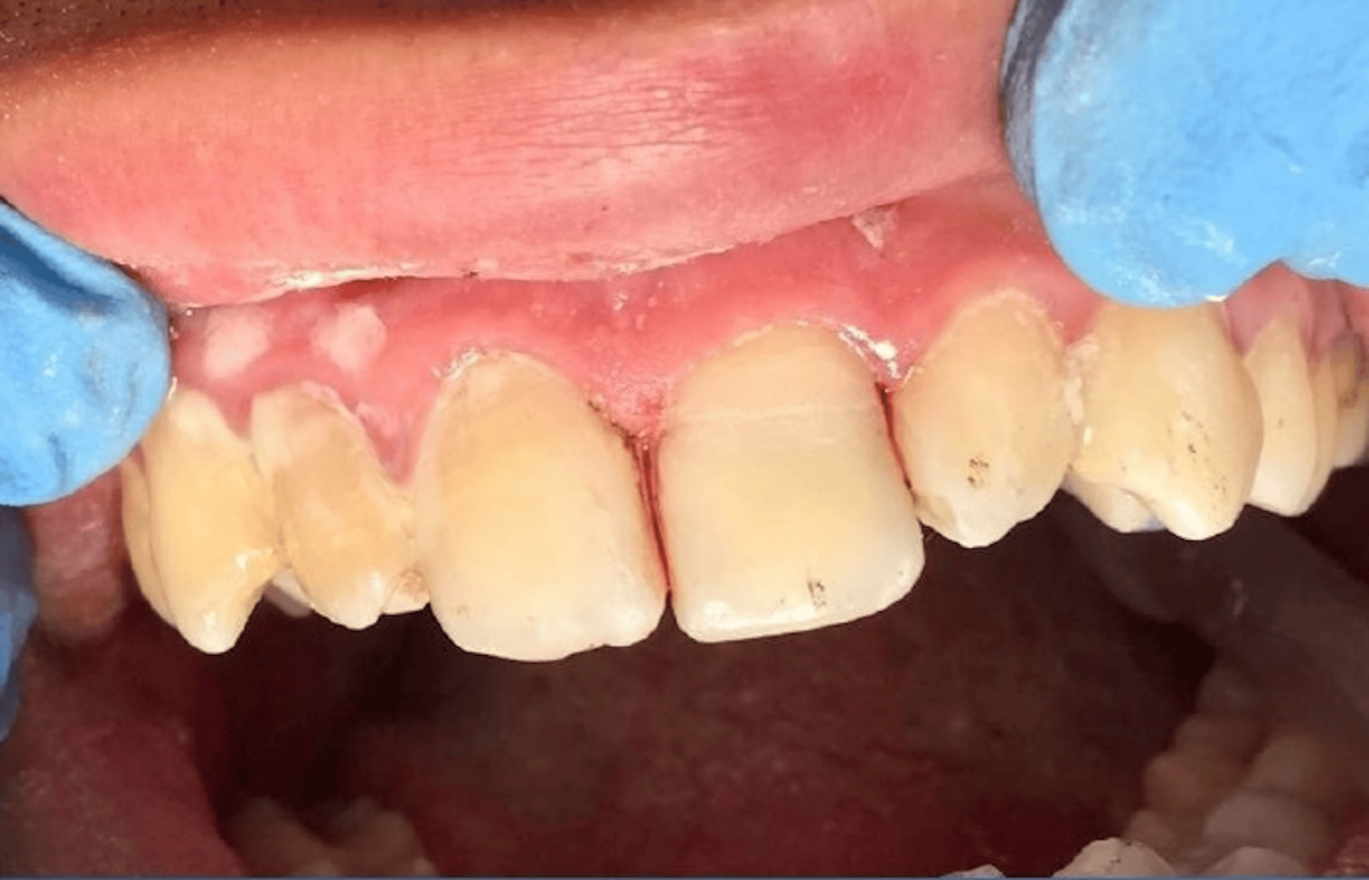
Reattachment of the coronal fragment The internal cavity of the crown fragment was prepared to accommodate the fiber post. Adhesive protocol was applied, and the fragment was reattached using the fiber post for reinforcement.

A cavity was created on the internal surface of the extracted coronal fragment to accommodate the fiber post. The same adhesive protocol was applied to both the post space and the fragment’s cavity. The fragment was repositioned and bonded to the remaining tooth structure with the support of the fiber post, resulting in a stable and esthetic reattachment (Figure 7).

**Figure 8:**
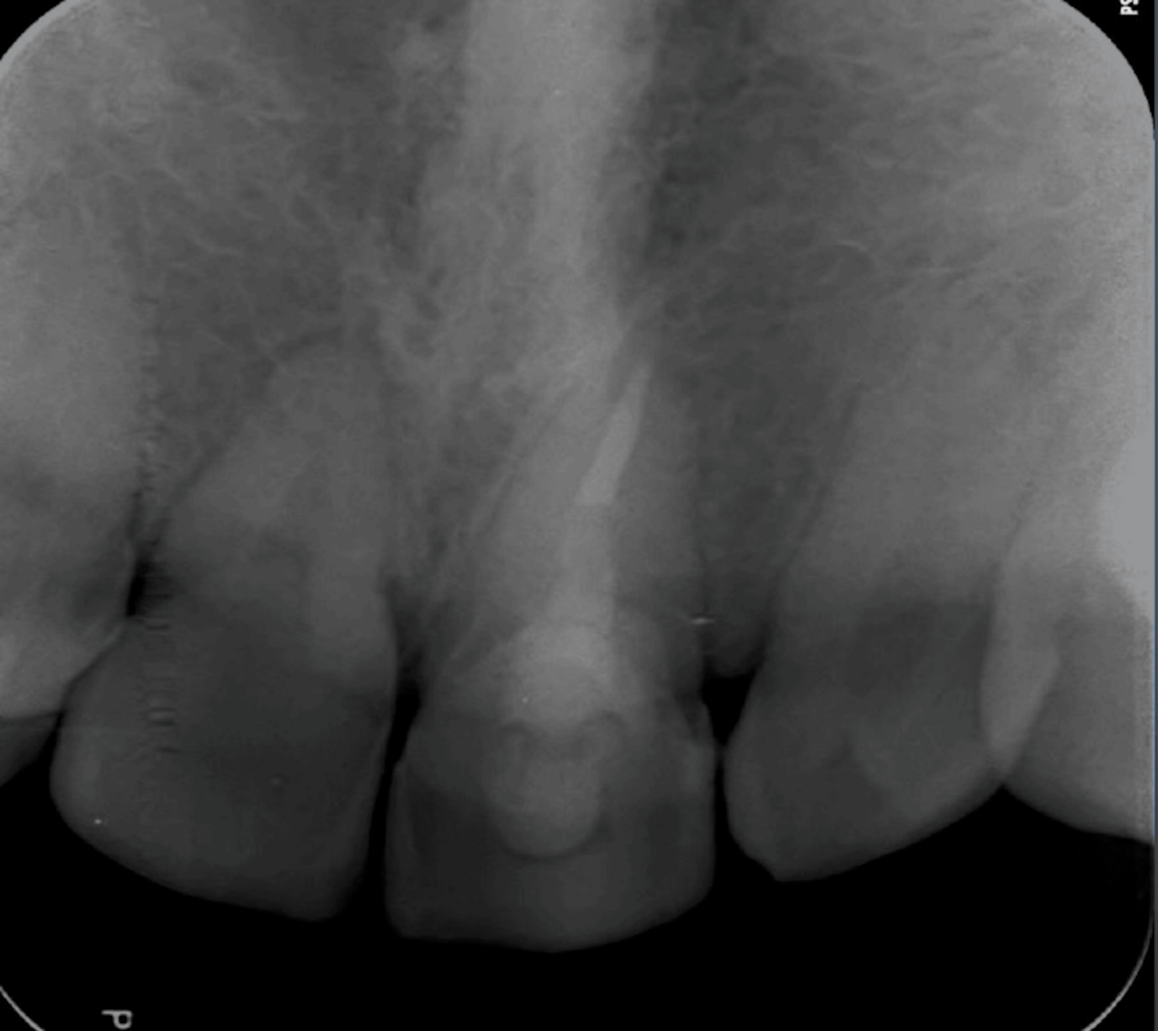
Final radiographic image Postoperative periapical radiograph showing proper adaptation of the fiber post and reattached coronal fragment.

A final periapical radiograph confirmed satisfactory obturation and accurate post placement (Figure 8). Although long-term follow-up could not be obtained due to the patient completing military service and relocating, verbal follow-up several months later confirmed that the tooth remained asymptomatic, functional, and esthetically acceptable.

## Discussion

Complicated crown-root fractures are among the most challenging traumatic dental injuries to manage, particularly when they affect the aesthetic zone. These types of fractures often extend subgingivally or even infrabony, complicating the restorative prognosis and requiring a multidisciplinary approach for optimal results [6].

In the present case, the tooth was provisionally diagnosed as an uncomplicated crown fracture, which highlights the diagnostic difficulty in detecting subgingival or root-level extensions without clinical exploration or removal of the coronal fragment. The definitive diagnosis was possible only after fragment removal and periodontal probing, which revealed a physiological probing depth of 2 mm on the palatal aspect.

Alternative treatment options for such cases include surgical crown lengthening [7], orthodontic extrusion [8], and extraction followed by prosthetic rehabilitation or implant placement [9]. These methods may be indicated when the fracture extends deeply subgingivally or when periodontal health is compromised. In this case, the limited subgingival extension, healthy periodontal status, and patient’s preference for a conservative approach allowed for successful management through fragment reattachment supported by a fiber post.

Fiber post-supported reattachment offers the advantage of preserving natural esthetics and functional integrity while being minimally invasive [10]. Fiber posts have an elastic modulus similar to dentin, distributing stress more evenly and reducing the risk of root fracture compared to metallic posts [11]. Literature reports have demonstrated favorable survival rates for reattached fragments when combined with fiber post reinforcement [3,12].

Allowing a healing period between the initial trauma and final restoration also contributed to a favorable outcome. In this case, an 11-day interval enabled soft tissue healing and ensured better reattachment stability. Although radiographic follow-up was not possible due to the patient’s relocation, verbal feedback indicated satisfactory function and esthetics.

Key clinical lessons

Key clinical lessons include the following: 1) Removal of the coronal fragment is essential to accurately determine the extent of a fracture; 2) Fiber post-supported reattachment can provide durable esthetic and functional outcomes in select cases; and 3) Conservative approaches should be prioritized when periodontal involvement is minimal and patient cooperation is high.

## Conclusions

This case report highlights the feasibility and success of a conservative approach in managing a complicated crown-root fracture in the aesthetic zone. The combination of root canal therapy, fiber post reinforcement, and fragment reattachment provided a biologically respectful and esthetically pleasing outcome without the need for surgical intervention.

Critical to success were the accurate diagnosis following removal of the coronal fragment, proper case selection, and appropriate timing of the definitive restoration. The preservation of the patient’s natural tooth structure not only met functional demands but also had positive psychological implications, especially considering the patient’s young age and social context.

While long-term follow-up was not possible due to the patient’s relocation after military service, the favorable short-term clinical outcome and patient satisfaction support the effectiveness of this minimally invasive treatment strategy. This case underscores the importance of considering conservative treatment options before opting for more invasive alternatives in cases of complicated crown-root fractures.
